# Quality of Life in Bipolar Type I Disorder in a One-Year Followup

**DOI:** 10.1155/2012/860745

**Published:** 2012-12-27

**Authors:** Homayoun Amini, Vandad Sharifi

**Affiliations:** ^1^Department of Psychiatry, Roozbeh Hospital, South Kargar Avenue, Tehran 1333795914, Iran; ^2^Psychiatry and Psychology Research Center, Tehran University of Medical Sciences, Tehran 1333795914, Iran

## Abstract

*Objectives*. The aims of this study were (i) to compare Quality of Life (QOL) of patients with bipolar disorder (BD) type I to those with schizophrenia during a one-year period after hospitalization and (ii) to assess the association of different domains of QOL with severity of clinical symptoms and level of functioning in bipolar patients group. *Method*. A hundred and two participants were consecutively recruited before discharge from an acute hospitalization. To measure QOL as the main outcome variable, the Farsi (Persian) version of the World Health Organization's QOL Instrument Short Version (WHOQOL BREF) was used. Affective symptoms, overall functioning, and severity of mental illness were assessed as well. The assessment procedure was repeated four, eight, and 12 months after discharge. *Results*. No significant differences were found between patients with BD and schizophrenia on four domains of WHOQOL BREF at the baseline and the four, eight, and 12 month assessments. Within the subjects with bipolar I disorder, the most stable finding was negative association of depression severity with WHOQOL-BREF on the all four domains during repeated assessments. *Conclusion*. The findings suggest that persistent depressive symptoms might be the primary determinant of impaired QOL in patients with bipolar I disorder.

## 1. Introduction 

Bipolar disorder (BD) is one of the most important leading causes of disability worldwide among young adults [1]. There is considerable literature on the clinical course of BD as well as the safety and efficacy of various treatments while the literature on their Quality Of Life (QOL) is quite sparse [2]. QOL is a multidimensional concept that emphasizes an individual's satisfaction with all aspects of life and includes physical, social, environmental, and psychological well-being [3]. While consistent assessment is lacking on QOL and levels of functioning in patients with bipolar disorder, even less information is available regarding the impact of treatment on these outcomes [2]. Although major determinants of subjective QOL in people with a severe mental illness are clinical features such as symptoms of depression and anxiety [4] but sole reliance on symptomatic outcome measures may not detect these more subtle changes in well-being, functioning, and QOL [5]. 

Patients with BD consistently had lower scores on QOL and functionality than did comparator groups no matter what instruments were used; these results indicate that the reduced Health Related QOL (HRQOL) and impairment of this disorder has a pernicious course even in the absence of active symptoms [2, 5–11]. The studies regarding to comparison of QOL of BD with schizophrenia show some conflicting results. Several studies showed relatively better QOL in BD compared with schizophrenia [12–15], while other studies reported that the patients with BD have significantly lower of QOL compared with the patients with schizophrenia [16, 17]. To decrease affective and/or cognitive bias, most of the studies on QOL in BD were conducted with post-hospitalized euthymic bipolar patients in developed countries. Regarding to this fact that patients with bipolar I disorder are non-euthymic more than 40% of weeks, [18] it is necessary to examine QOL in noneuthymic bipolar patients in a longitudinal research [5] especially in developing countries. 

The study aimed (i) to compare QOL between the patients with BD type I and patients with schizophrenia as a prototype of severe mental illness, during a one-year period after hospitalization and (ii) to assess the association of different domains of QOL with severity of clinical symptoms and level of functioning in bipolar patients group. 

## 2. Subjects and Methods

One hundred and two subjects participated in the study that included a cohort of 59 patients with BD type I, and a cohort of 43 patients with schizophrenia, 15 years or older, consecutively enrolled from April 2008 to November 2009. The participants were recruited from Roozbeh Hospital, a referral teaching hospital in Tehran, the capital of Iran, at the time of discharge after hospitalization due to an acute episode of mania, mixed, or psychosis. All subjects met the Diagnostic and Statistical Manual of Mental Disorders, Fourth edition (DSM-IV) criteria for bipolar I disorder or schizophrenia. Exclusion criteria included an inability to obtain informed consent or comply with study requirements. 

To measure QOL as the main outcome variable, the Farsi (Persian) version of the World Health Organization's QOL Instrument-Short Version (WHOQOL-BREF) [19, 20] was used. The WHOQOL-BREF is a self-administered, multidimensional scale used to assess physical, psychological, social, and environmental aspects of QOL. Also, we used the Young Mania Rating Scale (YMRS) [21] and the Hamilton Depression Rating Scale (HDRS) [22, 23] to assess manic and depressive symptoms, respectively. The Global Assessment of Functioning Scale (GAF), which is a clinician-rated composite measure of functioning and symptoms, was used to assess overall psychological, social, and occupational functioning in the past month [24, 25]. Additionally, we used the Clinical Global Impression-Severity (CGI-S) [26]. The CGI-S, a serial assessment, is also a clinician-rated scale to assess each patient's mental illness severity using a 1–7 response scale with higher scores corresponding to more severity. The assessment protocol was repeated 4-, 8-, and 12-months after hospital discharge. Inter-rater reliability on the YMRS, HDRS, GAF, and CGI-S scores was ascertained and intra-class correlation coefficients were between 0.7 and 0.85 across the above-mentioned scales.

Analyses were carried out in SPSS 16.0. Continuous and categorical variables were compared using the independent sample *t*-test (or Mann-Whitney test) and *χ* ^2^ test, respectively. For comparison of two groups in each assessment period, the ratings of WHOQOL-BREF were treated as continuous data and were analyzed by using a two-tailed, repeated measures analysis of variance (ANOVA), with the diagnosis as a between-subject grouping factor, the WHOQOL-BREF domain as within-subject grouping factor, and other variables including age, marital status, educational level, work status, and GAF as covariates. To check the assumptions for repeated measures ANOVA, we used Mauchly's Test of Sphericity, Levene's Test of Equality of Error Variances, and Box's Test of Equality of Covariance. As four WHOQOL-BREF domain scores in each assessment were correlated more than what we would expect by chance, we had to account for this correlation. We can say that the four domain scores come from the same “subject”, or that this measure was “repeated”. Therefore, we considered the WHOQOL-BREF domain as within-subjects factor. If a significant overall ANOVA was observed it would be followed by individual pair-wise comparisons that used a two-tailed Student's *t*-test corrected by the Bonferroni method for multiple comparisons. Within bipolar patients, we analyzed the association of clinical and functional variables with the domains of QOL at the baseline, 4-, 8-, 12-month assessments through multiple linear regressions. In order to check the assumptions for linear regression analysis, we used the lack of fit test, the Shapiro-Wilk test, and the Durbin-Watson statistic to test the assumption of linearity, of normality, and of independence of errors, respectively. Adjusted analyses were performed for each domain of the WHOQOL-BREF separately using stepwise linear regression models. The outcome variable in each model was the domain of the WHOQOL-BREF, treated as a separate numeric variable. The numerical YMRS, HDRS, GAF, and CGI scores were included as independent variables. In the adjusted analyses, the effect of the score of the WHOQOL-BREF was controlled for sex and age. Spearman correlation coefficients were calculated between each item of the HDRS and the WHOQOL-BREF domains. The adjustment of multiple comparisons was applied and a *P* value of less than 0.05 was considered as statistically significant. The Ethics Committee of Tehran University of Medical Sciences gave ethical clearance. Written informed consent was collected before entering the study.

## 3. Results

Thirty-four (59.6%) of the patients with BD and 24 (61.5%) of those with schizophrenia were male, the mean age was 33.6 (SD = 12.4) and 40.4 (SD = 11.0) years, respectively. Baseline demographic and clinical characteristics of the participants are reported in Table 1. Forty-eight (81.4%) of the patients with BD and 34 (79.1%) of those with schizophrenia completed the 12-month assessment. Thirty-two (56.1%) patients with bipolar I disorder experienced manic or mixed exacerbations during the one year follow-up. 

### 3.1. Comparison of BD and Schizophrenia Groups

The two groups were compared regarding demographic and clinical variables. All characteristics were not significantly different between the two patient groups but for age. Regarding to the observed near significant differences in terms of educational level, marital status, working status, and GAF score, these variables as well as age were used as covariates to compare the results on WHOQOL-BREF domains scores of the two patient groups. No significant differences were found between the ratings of the patients with BD and schizophrenia on four domains of WHOQOL-BREF at the baseline and the 4-, 8-, 12-month assessments (Table 2). 

### 3.2. Association of Different Domains of WHOQOL-BREF with Severity of Clinical Symptoms and Level of Functioning in BD Group

Within the subjects with bipolar disorder, the most stable finding was negative association of HDRS with WHOQOL-BREF on the all four domains during repeated assessment. The linear regression model revealed that the physical domain was significantly predicted by HDRS score, with variances ranging from 11% to 52%, and from 9% to 50% when adjusted for the clinical and demographic variables (Table 3); the psychological domain was significantly predicted by HDRS score, with variances ranging from 17% to 47%, and from 15% to 44% when adjusted for the clinical and demographic variables (Table 4); the social domain was significantly predicted by HDRS score, with variances ranging from 15% to 25%, and from 13% to 23% when adjusted for the clinical and demographic variables (Table 5); and the environmental domain was significantly predicted by HDRS score, with variances ranging from 10% to 40%, and from 8% to 37% when adjusted for the clinical and demographic variables (Table 6). Age and GAF were not associated with WHOQOL-BREF domains. However, the other observed associations of WHOQOL-BREF domains with the other variables such as sex, YMRS, and CGI in the one assessment were not consistent throughout other follow-up assessments (Table 3 to Table 6). 

Also, to explore the most stable correlations, that is, those between the depressive symptoms and QOL domains, we calculated Spearman correlation coefficients between each item of the HDRS and the WHOQOL-BREF domains. Several HDRS items were inversely associated with the physical domain of WHOQOL-BREF in all four follow-up assessments, consisting of depressed mood (rho: −0.51 to −0.40, *P* value < 0.01), suicidal impulses (rho: −043 to −0.36, *P* value < 0.01), psychic anxiety (rho: −0.49 to −0.31, *P* value < 0.01), and general somatic (rho: −0.51 to −0.38, *P* value < 0.01). Furthermore, depressed mood (rho: −0.65 to −0.41, *P* value < 0.01), suicidal impulses (rho: −0.57 to −0.40, *P* value < 0.01), and *psychic anxiety *(rho: −0.39 to −0.27, *P* value < 0.05) were inversely associated with the psychological domain of WHOQOL-BREF in all four assessments. In addition, only suicidal impulses (rho: −0.48 to −0.35, *P* value < 0.05) was inversely associated with the social domain of WHOQOL-BREF in all four assessments. However, none of the HDRS items show any stable associations with the environmental domain of WHOQOL-BREF in all four follow-up assessments, but suicidal impulses (rho: −0.54 to −0.41, *P* value < 0.01) was inversely associated with in three of those assessments. 

## 4. Discussion

In a longitudinal study, we compared QOL between the patients with BD type I and those with schizophrenia during one-year after hospitalization. The two groups were comparable to all WHOQOL-BREF domains throughout 12-month follow-up period. This finding was in line with some previous studies that reported the patients with BD had similar levels of QOL as those patients with schizophrenia [27–30]. However, the assessment of QOL in BD versus schizophrenia has provided some conflicting evidence. On the one hand, relatively better QOL was found in several studies that directly compared BD with schizophrenia, [12–15] with QOL of BD patients lying on a continuum between those of patients with schizophrenia and healthy subjects. On the other hand, there are some reports that the patients with BD have significantly lower scores of QOL compared with the patients with schizophrenia [16, 17]. The comparable QOL in two groups may be related to similar aspects of these chronic mental illnesses such as substantial disability, cognitive impairment, depressive symptoms, the adverse effects of therapy, and social stigma [27–29], particularly taking into account the features of our sample who were selected from patients with severe illness admitted in a referral hospital; however, we could not assess all of these features in the present study. 

Within the subjects with bipolar disorder, the most consistent finding was inverse association of HDRS with the physical and psychological domains of WHOQOL-BREF in the all four assessment times. The different WHOQOL-BREF domains were significantly predicted by HDRS score, with variances ranging from 10% to 52%, and from 8% to 50% when the clinical and demographic variables were added to the model. Özer and colleagues (2002) reported that only 13% of the observed variance of QOL score was predicted by the current depression subscale of the SADS interview while none of the historical demographic or clinical variables were predictive of QOL [31]. Also, Dias and colleagues found that depressive symptoms were strong predictors of physical, psychological, and environmental QOL [32]. In addition, it has been shown that eight of the nine QOL dimensions were significantly correlated with HDRS scores by using other instruments to measure QOL [33, 34]. The results of several studies suggest that persistent depressive symptoms are the primary determinant of impaired QOL in BD and these symptoms have been associated with more impairment in job, family, and social life [29, 35–38]. On the other hand, one study indicated that low QOL scores at the first visit can predict recurrence of depression in patients with BD [39]. Among various depressive symptoms of HDRS, “suicidal impulses” had the most stable association with the different domains of WHOQOL-BREF through the assessment period. In addition, the “psychic anxiety” symptom was inversely associated with the physical and the psychological domains of WHOQOL-BREF. Although we did not examine comorbidity with anxiety disorders but the negative association of psychic anxiety with QOL may be interpreted in line with previous reports indicating a detrimental effect of such comorbidity on QOL [40–42]. Comorbidity of BD and anxiety may be associated with markers of illness severity such as number of suicide attempts [43]. The nature of this negative association needs to be explored in the future studies. 

Regarding to the fact that the patients with BD type I experience depressive symptoms for about 30% of weeks, compared with about 10% of weeks for hypomanic or manic symptoms [18], it is necessary to manage depressive symptoms better to improve QOL more. 

In the present study, age was not associated with any domains of WHOQOL-BREF through the assessment period. The results of previous studies concerning the association of age and QOL were controversial [13, 44]. The result of present study did not show any association between WHOQOL-BREF and GAF score. This finding was in contrast with the finding of MacQueen et al. (2000) study that reported a strong correlation between the subjectively rated HRQOL and objectively rated GAF scores [10]. However, Goldberg and Harrow showed that subjectively assessed life satisfaction and objectively measured functioning may not be equivalent in more severely ill affective disorder patients [16, 45]. The association of the YMRS with WHOQOL-BREF scores in the one assessment was not consistent through the other assessment times. Tsevat et al. [46] reported that QOL measures did not relate to levels of mania; however, another study found the score of YMRS was negatively associated with the physical, psychological, and social domains of the WHOQOL [47]. 

The results of the present study should be interpreted in view of several limitations. The limited sample size of the study might have impeded to detect differences in WHOQOL-BREF scores between patients with BD and patients with schizophrenia. The study sample is not representative because the subjects were only recruited from one referral center. Bipolar group participants were restricted to whom affected by type I disorder and admitted to the hospital. Therefore, the results of the study cannot be generalized to all patients with bipolar disorder. Also, we used a generic instrument to examine QOL in patients with BD; however, using a condition-specific instrument could take into account aspects of the disorder that were more relevant [48]. Regarding to missing data due to incompleteness of WHOQOL-BREF on all four assessments, we were unable to use repeated measures analysis of variance (ANOVA), with the time as within-subject grouping factor. Nevertheless, the present study is a longitudinal study on QOL of bipolar patients with different severity of affective symptoms from a developing country. 

## 5. Conclusion

With regard to paucity of longitudinal data concerning the temporal relationship between QOL and clinical characteristics of patients with bipolar disorder, the consistent inverse association of depressive symptoms with various domains of QOL observed in this suggests more consideration in clinical practice and future research. In conclusion, the results of present study suggest that persistent depressive symptoms might be the primary determinant of impaired QOL in patients with bipolar I disorder. 

## Figures and Tables

**Table 1:** Demographic and clinical characteristics of patients with bipolar disorder (*N* = 59) or schizophrenia (*N* = 43).

Variable	Bipolar disorder *N* (%) or mean ± SD	Schizophrenia *N* (%) or mean ± SD	Test	*P* value
Gender (male)	34 (59.6)	24 (61.5)	*χ* ^2^ = 0.035	0.853
Age	33.6 ± 12.4	40.4 ± 11.0	*T* = −2.759	0.007**
Number of previous episodes	2.9 ± 2.4	4.6 ± 6.6	*Z* = −0.763	0.445
Education level			*χ* ^2^ = 13.383	0.063
Elementary school	11 (19.3)	5 (12.8)		
Middle school	24 (42.1)	7 (17.9)		
High school	11 (19.3)	9 (23.1)		
Graduate school	10 (17.5)	12 (30.8)		
University	1 (1.80)	6 (15.4)		
Marital status			*χ* ^2^ = 7.891	0.005**
Married	20 (35.7)	4 (10.3)		
Not married	36 (64.3)	35 (89.7)		
Working status			*χ* ^2^ = 2.594	0.107
Employed	9 (15.8)	2 (5.1)		
Not employed	48 (84.2)	37 (94.9)		
CGI	4.59 (1.2)	4.77 (1.0)	*T* = −0.771	0.443
GAF	31.36 (12.1)	27.05 (9.7)	*T* = 1.832	0.07

CGI: Clinical Global Impression score.

GAF: Global Assessment of Functioning score.

**Table 2:** Comparison of bipolar disorder and schizophrenia in terms of the WHOQOL-BREF domains scores at discharge, 4-, 8-, 12-month assessments.

Time/Domain	Bipolar disorder mean ± SD	Schizophrenia mean ± SD	*F*	*P* value	*T*	*P* value
Discharge/Domain			1.21	0.275		
Physical	79.43 ± 16.79	72.02 ± 16.44			1.911	0.059
Psychological	69.58 ± 23.52	65.49 ± 20.46			0.931	0.354
Social	61.11 ± 23.89	61.22 ± 21.86			0.111	0.912
Environmental	62.19 ± 19.29	60.95 ± 16.72			0.386	0.700
4-month/Domain			0.58	0.450		
Physical	64.96 ± 19.43	65.61 ± 19.91			−0.325	0.746
Psychological	52.70 ± 21.41	60.34 ± 19.59			−1.494	0.140
Social	54.73 ± 21.56	60.19 ± 24.50			−1.064	0.291
Environmental	57.30 ± 18.04	58.47 ± 19.61			−0.315	0.754
8-month/Domain			0.02	0.889		
Physical	65.48 ± 22.03	64.47 ± 21.44			0.111	0.912
Psychological	59.72 ± 20.31	56.84 ± 21.52			0.410	0.684
Social	54.63 ± 21.40	60.53 ± 24.19			−0.848	0.400
Environmental	54.43 ± 21.36	59.61 ± 23.20			−0.875	0.386
12-month/Domain			1.01	0.321		
Physical	51.84 ± 17.20	51.40 ± 17.88			0.148	0.883
Psychological	50.24 ± 15.49	51.09 ± 14.82			−0.269	0.789
Social	54.52 ± 27.22	53.99 ± 24.21			0.160	0.874
Environmental	57.38 ± 19.78	55.11 ± 21.62			0.413	0.681

*Repeated Measures Analysis of Variance (ANOVA).

**Independent Student *T*-Test.

**Table 3:** Correlation coefficients of the physical domain scores of WHOQOL-BREF with the severity of clinical symptoms and level of functioning in bipolar disorder group at baseline, 4-, 8-, 12-month assessments.

Assessment	Model		Unstandardized coefficients		Standardized coefficients	*T*	*P* value	*R*	*R* square	Adjusted *R* square	Collinearity statistics (VIF)
			*B*	St. Er	Beta						
Baseline (*N* = 57)	1	HDRS	−1.87	0.35	−0.59	−5.42	<0.001	0.59	0.35	0.34	1.00
	2	HDRS	−2.11	0.33	−0.67	−6.41	<0.001	0.67	0.45	0.43	1.06
		CGI	5.30	1.70	0.33	3.12	<0.01				1.06

4-month (*N* = 48)	1	HDRS	−0.16	0.07	−0.33	−2.36	<0.05	0.33	0.11	0.09	1.00

8-month (*N* = 44)	1	HDRS	−1.72	0.35	−0.61	−4.97	<0.001	0.61	0.37	0.36	1.00
	2	HDRS	−2.19	0.33	−0.77	−6.61	<0.001	0.72	0.52	0.50	1.18
		YMRS	0.99	0.27	0.43	3.63	<0.001				1.18

12-month (*N* = 45)	1	HDRS	−0.97	0.27	−0.48	−3.60	<0.001	0.48	0.23	0.21	1.00
	2	HDRS	−1.17	0.25	−0.58	−4.67	<0.001	0.62	0.39	0.37	1.07
		Sex	−13.93	4.29	−0.41	−3.25	<0.01				1.07

HDRS: Hamilton-Depression Rating Scale.

CGI: Clinical Global Impression.

YMRS: Young Mania Rating Scale.

**Table 4:** Correlation coefficients of the psychological domain scores of WHOQOL-BREF with the severity of clinical symptoms and level of functioning in bipolar disorder group at baseline, 4-, 8-, 12-month assessments.

Assessment	Model		Unstandardized coefficients		Standardized coefficients	*T*	*P* value	*R*	*R* square	Adjusted *R* square	Collinearity statistics (VIF)
			*B*	St. Er	Beta						
Baseline (*N* = 57)	1	HDRS	−1.62	0.49	−0.41	−3.30	<0.01	0.41	0.17	0.15	1.00
	2	HDRS	−1.80	0.45	−0.45	−3.98	<0.001	0.56	0.31	0.29	1.01
		YMRS	0.88	0.26	0.38	3.38	<0.001				1.01

4-month (*N* = 48)	1	HDRS	−1.38	0.39	−0.47	−3.58	<0.001	0.47	0.22	0.20	1.00

8-month (*N* = 44)	1	HDRS	−1.65	0.39	−0.55	−4.28	<0.001	0.55	0.30	0.29	1.00
	2	HDRS	−2.16	0.37	−0.72	−5.84	<0.001	0.68	0.47	0.44	1.18
		YMRS	1.09	0.31	0.44	3.56	<0.001				1.18

12-month (*N* = 45)	1	HDRS	−1.01	0.24	−0.54	−4.24	<0.001	0.54	0.29	0.28	1.00

HDRS: Hamilton-Depression Rating Scale.

YMRS: Young Mania Rating Scale.

**Table 5:** Correlation coefficients of the social domain scores of WHOQOL-BREF with the severity of clinical symptoms and level of functioning in bipolar disorder group at baseline, 4-, 8-, 12-month assessments.

Assessment	Model		Unstandardized coefficients		Standardized coefficients	*T*	*P* value	*R*	*R* square	Adjusted *R* square	Collinearity statistics (VIF)
			*B*	St. Er	Beta						
4-month (*N* = 47)	1	YMRS	−0.652	0.27	−0.34	−2.41	<0.05	0.34	0.11	0.09	1.00

8-month (*N* = 44)	1	HDRS	−1.44	0.39	−0.50	−3.71	<0.001	0.50	0.25	0.23	1.00
	2	HDRS	−1.51	0.37	−0.52	−4.13	<0.001	0.59	0.35	0.32	1.01
		Sex	−14.97	5.85	−0.32	−2.56	<0.05				1.01

12-month (*N* = 45)	1	HDRS	−1.13	0.42	−0.38	−2.72	<0.01	0.38	0.15	0.13	1.00

YMRS: Young Mania Rating Scale.

HDRS: Hamilton-Depression Rating Scale.

**Table 6:** Correlation coefficients of the environmental domain scores of WHOQOL-BREF with the severity of clinical symptoms and level of functioning in bipolar disorder group at baseline, 4-, 8-, 12-month assessments.

Assessment	Model		Unstandardized coefficients		Standardized coefficients	*T*	*P* value	*R*	*R* square	Adjusted *R* square	Collinearity statistics (VIF)
			*B*	St. Er	Beta						
Baseline (*N* = 56)	1	HDRS	−1.00	0.41	−0.31	−2.42	<0.05	0.31	0.10	0.08	1.00

4-month (*N* = 47)	1	YMRS	−0.69	0.25	−0.38	−2.73	<0.01	0.38	0.14	0.12	1.00
	2	YMRS	−0.69	0.24	−0.37	−2.81	<0.01	0.47	0.22	0.19	1.00
		Sex	−9.68	4.55	−0.28	−2.13	<0.05				1.00

8-month (*N* = 44)	1	HDRS	−1.36	0.36	−0.54	−4.19	<0.001	0.54	0.30	0.29	1.00
	2	HDRS	−1.42	0.30	−0.57	−4.69	<0.001	0.63	0.40	0.37	1.01
		Sex	−13.19	4.86	−0.33	−2.72	<0.05				1.01

12-month (*N* = 44)	1	HDRS	−0.99	0.33	−0.42	−2.96	<0.01	0.42	0.17	0.15	1.00

HDRS: Hamilton-Depression Rating Scale.

YMRS: Young Mania Rating Scale.
